# Supplementary material for: The Potential Effect of General Anesthetics in Cancer Surgery: Meta-Analysis of Postoperative Metastasis and Inflammatory Cytokines
Source: Cancers (Basel). 2023 May 15;15(10):2759. doi: 10.3390/cancers15102759 (PMC10216624; doi:10.3390/cancers15102759)
Supplement: Supplementary file 1 [file cancers-15-02759-s001.zip › Supplementary File.pdf]

SUPPLEMENTARY TABLE 1. CHARACTERISTICS OF INCLUDED TRIALS FOR LONG-TERM CANCER OUTCOMES

	Methods	Cancer type	Participants # (matched #)	Follow-up time (year)	Anesthesia		Outcome	Notes
					Propofol-based TIVA	Volatile anesthesia		
**HUANG 2019**	Single center Retrospective	Breast cancer	976(888)	5	Propofol; fentanyl	Desflurane; fentanyl	Metastasis;OS	Propensity matched
**HUANG 2020**	Single center Retrospective	Gastric cancer	408(334)	5	Propofol; fentanyl	Desflurane; fentanyl; propofol	Metastasis;OS	Propensity matched
**HUANG 2021**	Single center Retrospective	Glioblastoma	103(76)	2.5	Propofol; fentanyl	Desflurane; fentanyl	OS; recurrence	Propensity matched
**KOO 2020**	Single center Retrospective	Hepatocellular carcinoma	259	2	Propofol; remifentanil	Inhaled anesthsia; remifentanil	recurrence	

**LAI 2019**	Single center Retrospective	Hepatocellular carcinoma	944 (670)	10	Propofol; fentanyl	Desflurane; fentanyl	Metastasis;OS	Propensity matched
**LAI 2019**	Single center Retrospective	Intrahepatic cholangiocarcinoma	70 (58)	10	Propofol; fentanyl	Desflurane; fentanyl	Metastasis;OS	Propensity matched
**LAI 2020**	Single center Retrospective	Prostate cancer	631(528)	10	Propofol; fentanyl	Desflurane; fentanyl	OS	Propensity matched
**LAI 2020**	Single center Retrospective	Pancreatic cancer	140 (116)	10	Propofol; fentanyl	Desflurane; fentanyl	OS; recurrence	Propensity matched
**LEE 2016**	Single center Retrospective	Breast cancer	325	5	Propofol; opioid	Sevoflurane; opioid	OS; recurrence	
**MIAO 2022**	Single center Retrospective	Oral cancer	1347(604)	5	Propofol-based TIVA	Sevoflurane	OS; recurrence	Propensity matched
**PFAIL 2020**	Single center	Bladder cancer	231	3	Propofol	Inhaled anesthetics	OS; recurrence	

	Retrospective							
**SCHMOCH 2021**	Single center Retrospective	Glioma	471 (144)	6	Propofol	Sevoflurane	OS; recurrence	1:2 Propensity matched
**SESSLER 2019**	Multiple center RCT	Breast cancer	2132 (2108)	3	Paravertebral blocks; propofol	Sevoflurane opioid	Recurrence	
**SHIONO 2020**	Single center Retrospective	Breast cancer	1026 (308)	1	Propofol; remifentanil	Sevoflurane; remifentanil	recurrence	Propensity matched
**TAKEYAMA 2021**	Single center Retrospective	Gynecologic cancer	287 (188)	10	Propofol	Sevoflurane	OS; recurrence	Propensity matched
**YAN 2018**	RCT, blinding not clear	Breast cancer	80	2	Propofol; fentanyl	Sevoflurane; fentanyl	OS; recurrence;	
**YAN 2019**	RCT	Breast cancer	80	2	TIVA; fentanyl	sevoflurane; fentanyl	OS; recurrence	

**YOO 2019**	Single center Retrospective	Breast cancer	5331 (3532)	5	Propofol; remifentanil	Inhaled anesthetics	OS; recurrence	Propensity matched
**SUN 2022**	Single center Retrospective	Osteosarcoma	56(44)	4	Propofol	Desflurane	OS; recurrence	Propensity matched
**ZHANG 2021**	Single center Retrospective	Breast cancer	2760	5	Paravertebral blocks; propofol	Sevoflurane	Mortality; recurrence	Propensity matched

OS: overall survival;

SUPPLEMENTARY TABLE 2. CHARACTERISTICS OF INCLUDED TRIALS FOR POSTOPERATIVE CYTOKINES

**STUDY ID**	cancer type	Methods	Participants		Surgery duration (min with SD or range)		Outcomes	Note
			TIVA	INHA	TIVA	INHA		
**OH 2018**	breast cancer	RCT, observer blinded	99	102	110(85-133)	103(80-135)	IL-6/IL-10/IL-12/TGF-β	
**LIM 2018**	breast cancer	RCT, observer blinded	23	21	97±33	114±44	IL-6/IL-10/TNF-α	NCT02758249 on February 26, 2016.
**DEEGAN 2010**	breast cancer	RCT	15	17			IL-6/IL-10/TNF-α/IL-2/IL-4/IL-8/IL-1β/IL-12p70/IL-13/IFN-γ/MMP-1/MMP-3/MMP-9	

**JIN 2013**	lung cancer	RCT	20	20			IL-6/IL-10/TNF-α
**TIAN 2017**	lung cancer	RCT	31	31			IL-6/IL-10/MMP-9
**LEE 2012**	Esophegeal cancer	RCT	24	24	251.8 ± 68.0	289.1 ± 43.0	IL-6/CRP
**WAYAKABYASHI 2014**	Esophegeal cancer	RCT	24	24	422.7±35.4	389.5±59.6	IL-6/IL-8
**QIAO 2015**	Esophegeal cancer	double blind, randomized, controlled trial.	30	30			IL-6/TNF-α
**MARGARIT 2014**	colorectal cancer	RCT	30	30	130±40	125±39	IL-6/IL-10

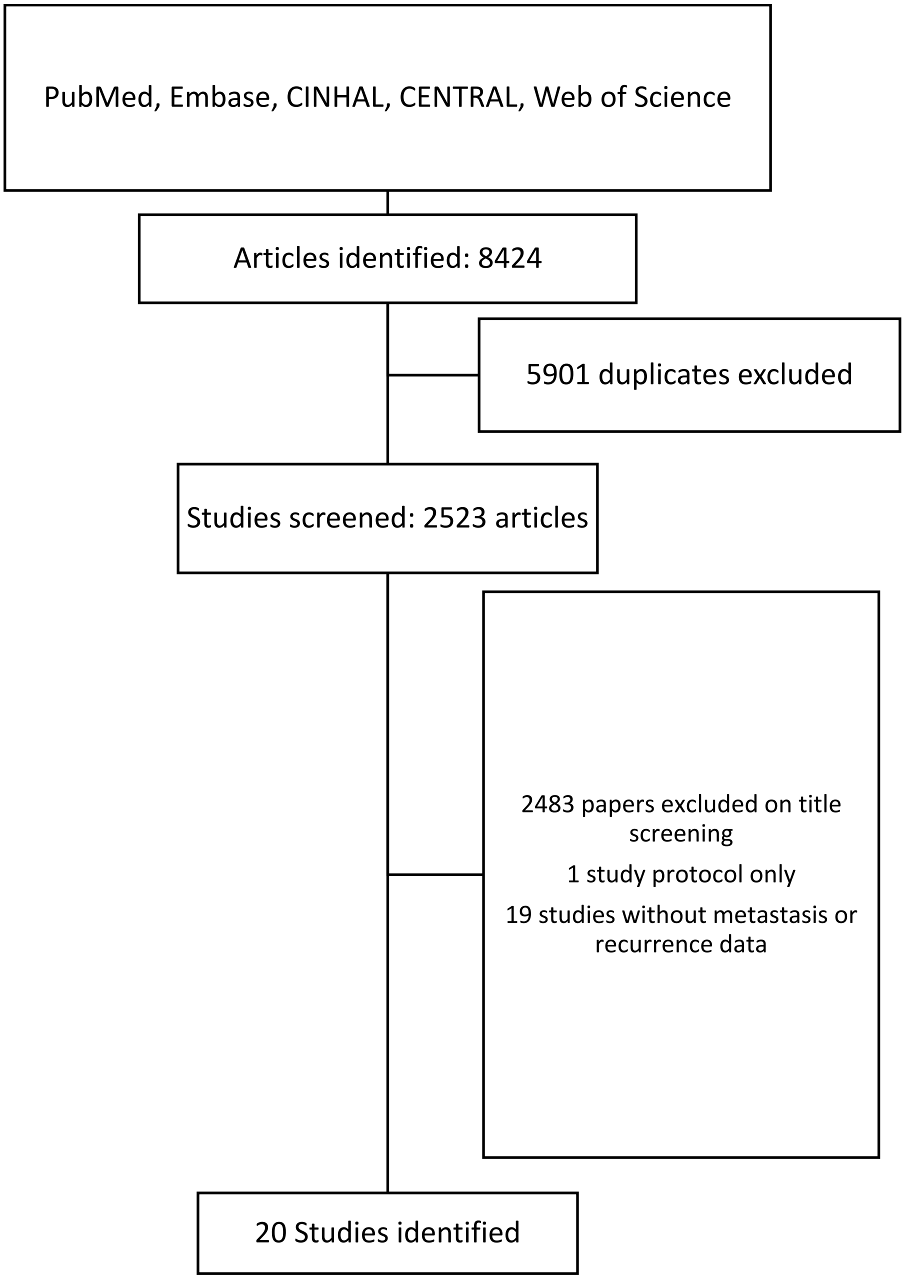

Search Strategy:
(survival [Mesh Terms]) OR (recurrence[Mesh Terms]) OR (mortality[Mesh Terms]) OR (progression[Mesh Terms]) OR (death[Mesh Terms]) OR (metastasis[Mesh Terms])
AND
((“General Analgesia”[Mesh Terms]) OR (“TIVA”[MeSH Terms]) OR (“total intravenous”[MeSH Terms]) OR(“propofol”[MeSH Terms]) OR (“volatile anesthesia”[MeSH Terms])OR (“inhaled anesthesia”[MeSH Terms])
And

((“cancer”[MeSH Terms]) OR (“Malignancy”[MeSH Terms]) OR (“neoplasm”[MeSH Terms]) OR (“tumor”[MeSH Terms]))

Supplementary Figure 1. Flow chart of the study selection for cancer long-term outcome under different general anesthesia and search criteria

	Study participant	Study attrition	Prognostic factor measurement	Outcome measurement	Study confounding	Statistical analysis &reporting
Huang 2019	?	?	+	+	+	+
Huang 2020	+	?	+	+	+	+
Huang 2021*	+	?	+	+	+	+
Koo 2020*	+	?	+	+	+	+
Lai (Hepatectomy) 2019	+	?	+	+	+	+
Lai (intrahepatic) 2019	+	?	+	+	+	+
Lai (Pancreatic) 2020	+	?	+	+	+	+
Lai (Prostatectomy) 2020*	+	?	+	+	+	+
Lee 2016	+	?	+	+	+	+
Miao 2022*	+	?	+	+	+	+
Pfail 2021	+	+	+	+	+	+
Schmoch 2021*	?	+	+	+	?	+
Sessler 2019*	+	+	+	+	+	+
Shiono 2020*	+	+	+	+	+	+
Sun 2022	+	?	+	+	+	+
Takeyama 2021*	+	?	+	+	+	+
Yan 2018*	+	?	+	+	?	+
Yan 2019	+	+	+	+	+	+
Yoo 2019*	+	+	+	+	?	+
Zhang 2021	+	+	+	+	+	+

Supplementary Figure 2. Risk of bias assessment summary of clinical studies for long-term cancer outcome

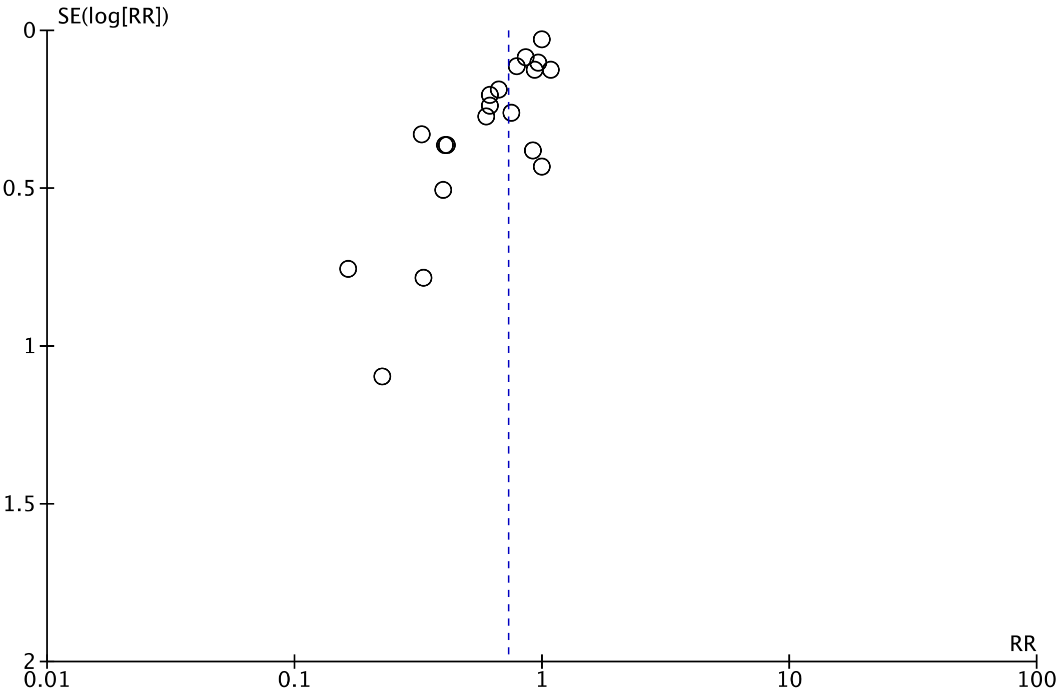

Supplementary Figure 3. Funnel plot of all included studies for publication bias analysis

Study or Subgroup	TIVA Events	TIVA Total	INHA Events	INHA Total	Weight	Risk Ratio M-H, Fixed, 95% CI
**5.4.1 Metastasis**						
Huang 2019	19	317	46	575	3.6%	0.75 [0.45, 1.26]
Huang 2020	34	155	55	167	5.8%	0.67 [0.46, 0.96]
Lai (Hepatectomy) 2019	25	313	43	331	4.6%	0.61 [0.39, 0.98]
Lai (intrahepatic) 2019	9	56	9	56	1.0%	1.00 [0.43, 2.33]
Lai (Pancreatic) 2020	7	29	17	29	1.9%	0.41 [0.20, 0.84]
Lee 2016	19	173	28	152	3.3%	0.60 [0.35, 1.02]
Pfail 2021	11	126	28	105	3.4%	0.33 [0.17, 0.63]
Sun 2022	4	22	10	22	1.1%	0.40 [0.15, 1.08]
Yan 2019	1	42	4	38	0.5%	0.23 [0.03, 1.94]
Zhang 2021	118	1395	150	1395	16.5%	0.79 [0.63, 0.99]
**Subtotal (95% CI)**		**2628**		**2870**	**41.7%**	**0.67 [0.58, 0.77]**
Total events	247		390			
Heterogeneity: Chi² = 11.72, df = 9 (P = 0.23); I² = 23%						
Test for overall effect: Z = 5.35 (P < 0.00001)						
**5.4.2 Recurrence**						
Huang 2021*	31	38	36	38	4.0%	0.86 [0.73, 1.02]
Koo 2020*	26	121	48	138	4.9%	0.62 [0.41, 0.93]
Lai (Prostatectomy) 2020*	2	264	12	264	1.3%	0.17 [0.04, 0.74]
Miao 2022*	109	302	113	302	12.5%	0.96 [0.78, 1.19]
Schmoch 2021*	50	52	89	92	7.1%	0.99 [0.93, 1.06]
Sessler 2019*	102	1043	111	1065	12.1%	0.94 [0.73, 1.21]
Shiono 2020*	12	159	13	159	1.4%	0.92 [0.43, 1.96]
Takeyama 2021*	9	94	22	94	2.4%	0.41 [0.20, 0.84]
Yan 2018*	2	40	6	40	0.7%	0.33 [0.07, 1.55]
Yoo 2019*	118	1766	108	1766	11.9%	1.09 [0.85, 1.41]
**Subtotal (95% CI)**		**3879**		**3958**	**58.3%**	**0.90 [0.82, 1.00]**
Total events	461		558			
Heterogeneity: Chi² = 25.57, df = 9 (P = 0.002); I² = 65%						
Test for overall effect: Z = 1.96 (P = 0.05)						
**Total (95% CI)**		**6507**		**6828**	**100.0%**	**0.80 [0.74, 0.88]**
Total events	708		948			
Heterogeneity: Chi² = 78.34, df = 19 (P < 0.00001); I² = 76%						
Test for overall effect: Z = 5.05 (P < 0.00001)						
Test for subgroup differences: Chi² = 10.89, df = 1 (P = 0.0010), I² = 90.8%						

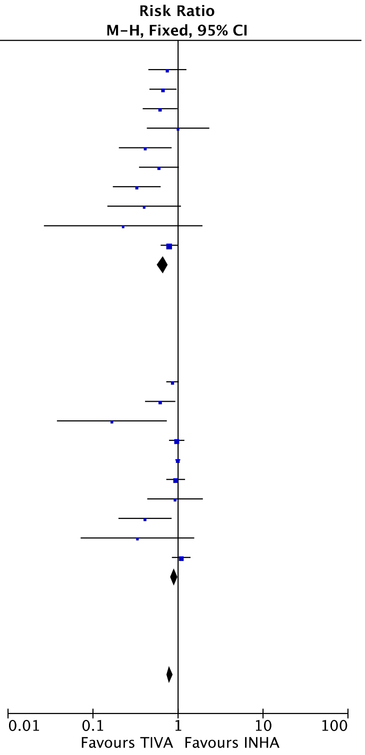

Supplementary Figure 4. Subgroup analysis of metastasis or recurrence in TIVA and IHNA cohorts

Study or Subgroup	TIVA Events	TIVA Total	INHA Events	INHA Total	Weight	Risk Ratio M-H, Fixed, 95% CI
**5.6.1 Breast cancer**						
Huang 2019	19	317	46	575	5.0%	0.75 [0.45, 1.26]
Lee 2016	19	173	28	152	4.6%	0.60 [0.35, 1.02]
Sessler 2019*	102	1043	111	1065	16.9%	0.94 [0.73, 1.21]
Shiono 2020*	12	159	13	159	2.0%	0.92 [0.43, 1.96]
Yan 2018*	2	40	6	40	0.9%	0.33 [0.07, 1.55]
Yan 2019	1	42	4	38	0.6%	0.23 [0.03, 1.94]
Yoo 2019*	118	1766	108	1766	16.6%	1.09 [0.85, 1.41]
Zhang 2021	118	1395	150	1395	23.1%	0.79 [0.63, 0.99]
**Subtotal (95% CI)**		**4935**		**5190**	**69.8%**	**0.87 [0.77, 0.99]**
Total events	391		466			
Heterogeneity: $Chi^2 = 9.43$, df = 7 (P = 0.22); $I^2 = 26\%$						
Test for overall effect: Z = 2.05 (P = 0.04)						
**5.6.2 Liver cancer**						
Koo 2020*	26	121	48	138	6.9%	0.62 [0.41, 0.93]
Lai (Hepatectomy) 2019	25	313	43	331	6.4%	0.61 [0.39, 0.98]
Lai (intrahepatic) 2019	9	56	9	56	1.4%	1.00 [0.43, 2.33]
**Subtotal (95% CI)**		**490**		**525**	**14.7%**	**0.65 [0.49, 0.87]**
Total events	60		100			
Heterogeneity: $Chi^2 = 1.11$, df = 2 (P = 0.57); $I^2 = 0\%$						
Test for overall effect: Z = 2.88 (P = 0.004)						
**5.6.3 Brain cancer**						
Huang 2021*	31	38	36	38	5.5%	0.86 [0.73, 1.02]
Schmoch 2021*	50	52	89	92	9.9%	0.99 [0.93, 1.06]
**Subtotal (95% CI)**		**90**		**130**	**15.4%**	**0.95 [0.88, 1.02]**
Total events	81		125			
Heterogeneity: $Chi^2 = 3.33$, df = 1 (P = 0.07); $I^2 = 70\%$						
Test for overall effect: Z = 1.50 (P = 0.13)						
**Total (95% CI)**		**5515**		**5845**	**100.0%**	**0.85 [0.77, 0.94]**
Total events	532		691			
Heterogeneity: $Chi^2 = 34.78$, df = 12 (P = 0.0005); $I^2 = 65\%$						
Test for overall effect: Z = 3.16 (P = 0.002)						
Test for subgroup differences: $Chi^2 = 6.54$, df = 2 (P = 0.04), $I^2 = 69.4\%$						

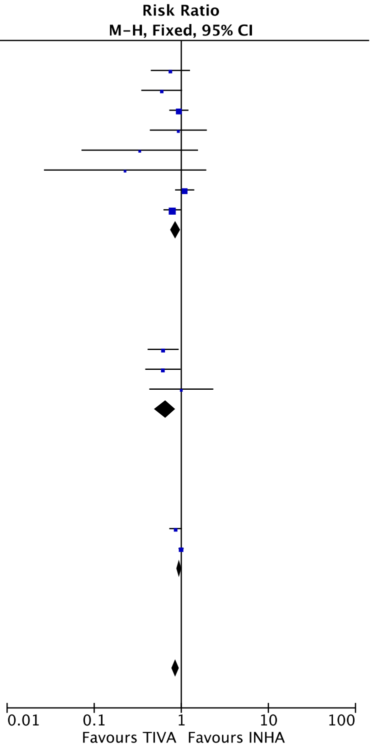

Supplementary Figure 5. Subgroup analysis of relative risk for metastasis or recurrence according to cancer types

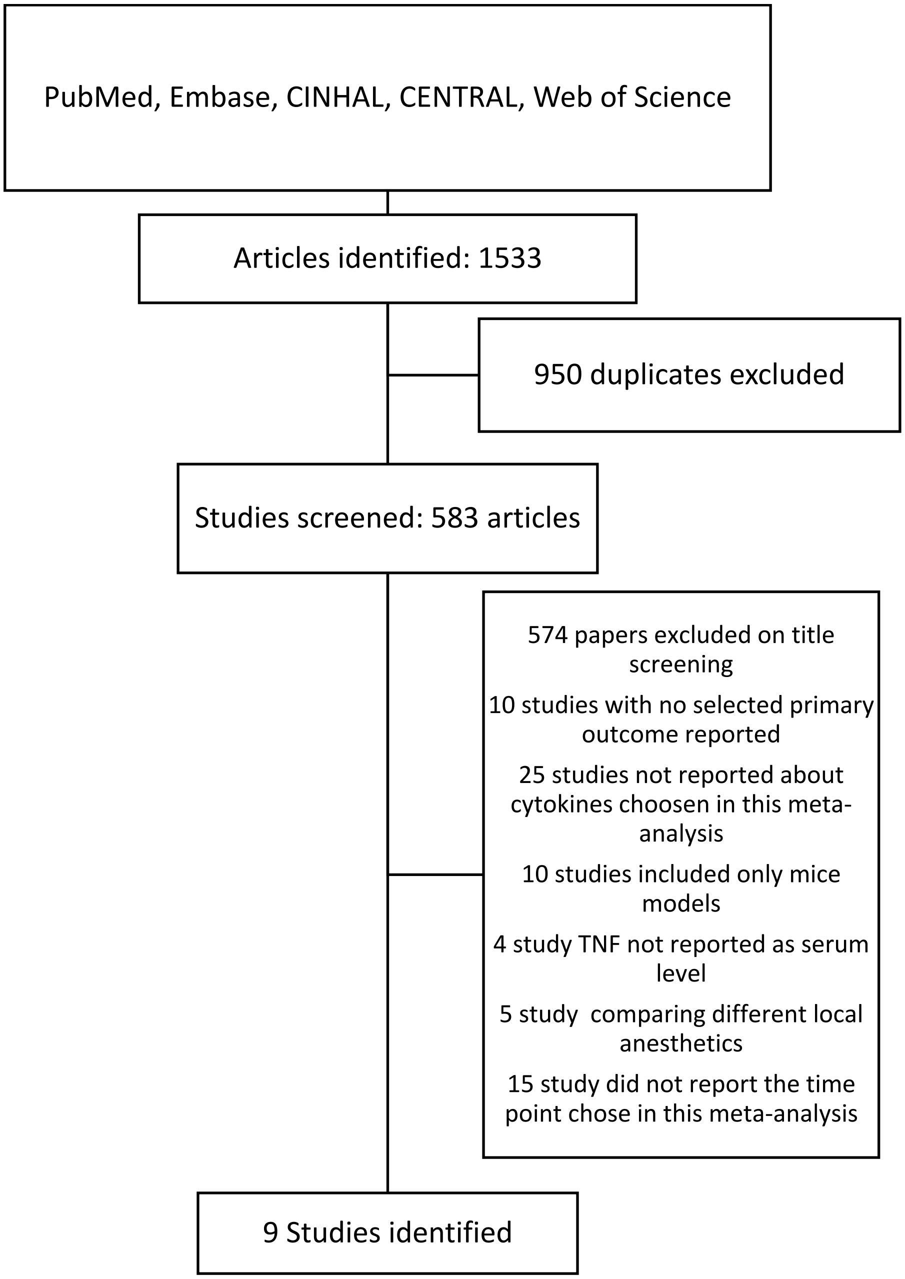

Search Strategy:

((“cytokine”[MeSH Terms]) OR (“cytokine”))OR ((“Interleukin”[MeSH Terms]) OR (“Interkeukin”))

AND

((“General Analgesia”[Mesh Terms]) OR (“TIVA”[MeSH Terms]) OR (“total intravenous”[MeSH Terms]) OR(“propofol”[MeSH Terms]) OR (“volatile anesthesia”[MeSH Terms])OR (“inhaled anesthesia”[MeSH Terms])

And

(("cancer"[MeSH Terms]) OR ("Malignancy"[MeSH Terms]) OR ("neoplasm"[MeSH Terms]) OR ("tumor"[MeSH Terms]))

Supplementary Figure 6. Flow chart of the study selection for inflammatory cytokines under different general anesthesia and search criteria

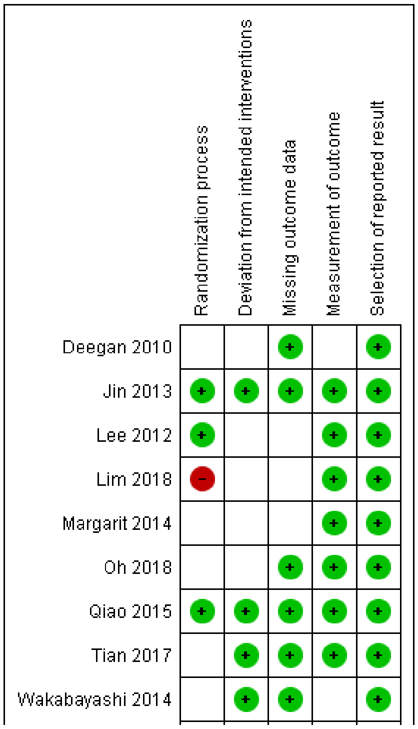

Supplementary Figure 7: Risk of bias summary for studies on postoperative cytokine release

Study or Subgroup	INHA Mean	INHA SD	INHA Total	TIVA Mean	TIVA SD	TIVA Total	Weight	Std. Mean Difference IV, Random, 95% CI
**1.2.1 breast cancer**								
Deegan 2010	8.025	1.675	17	10.975	4.24	15	12.4%	-0.91 [-1.65, -0.18]
Lim 2018	92.5	2.5	21	92.5	2.5	23	12.9%	0.00 [-0.59, 0.59]
Oh 2018	340	13.3	102	330	28.3	99	13.7%	0.45 [0.17, 0.73]
**Subtotal (95% CI)**			**140**			**137**	**38.9%**	**-0.09 [-0.84, 0.66]**
Heterogeneity: Tau² = 0.36; Chi² = 12.34, df = 2 (P = 0.002); I² = 84%								
Test for overall effect: Z = 0.24 (P = 0.81)								
**1.2.2 lung cancer**								
Jin 2013	16	5	20	15	4	20	12.8%	0.22 [-0.41, 0.84]
Tian 2017	56,440.9	6,677.2	31	48,126	4,913.4	31	13.0%	1.40 [0.84, 1.96]
**Subtotal (95% CI)**			**51**			**51**	**25.8%**	**0.82 [-0.34, 1.98]**
Heterogeneity: Tau² = 0.61; Chi² = 7.70, df = 1 (P = 0.006); I² = 87%								
Test for overall effect: Z = 1.38 (P = 0.17)								
**1.2.3 Esophegeal cancer**								
Lee 2012	183.897	100.637	24	184.07	71.978	24	13.0%	-0.00 [-0.57, 0.56]
Qiao 2015	121.556	7.333	30	94.667	5.778	30	11.7%	4.02 [3.12, 4.92]
Wakabayashi 2014	89.6	16.1	10	57.3	12.6	10	10.6%	2.14 [0.99, 3.29]
**Subtotal (95% CI)**			**64**			**64**	**35.3%**	**2.04 [-0.59, 4.66]**
Heterogeneity: Tau² = 5.16; Chi² = 57.50, df = 2 (P < 0.00001); I² = 97%								
Test for overall effect: Z = 1.52 (P = 0.13)								
**Total (95% CI)**			**255**			**252**	**100.0%**	**0.86 [0.09, 1.63]**
Heterogeneity: Tau² = 1.11; Chi² = 97.21, df = 7 (P < 0.00001); I² = 93%								
Test for overall effect: Z = 2.18 (P = 0.03)								
Test for subgroup differences: Chi² = 3.50, df = 2 (P = 0.17), I² = 42.8%								

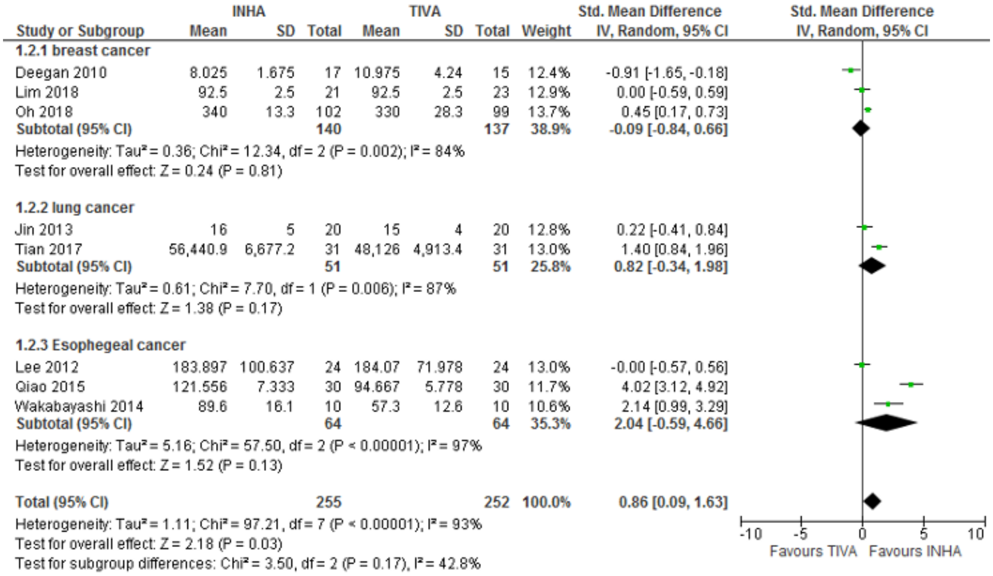

Supplementary Figure 8. Subgroup analysis of IL-6 at 24 hours after surgery

Study or Subgroup	INHA Mean	INHA SD	INHA Total	TIVA Mean	TIVA SD	TIVA Total	Weight	Std. Mean Difference IV, Random, 95% CI
Deegan 2010	3.9	0.5	17	4.225	0.2602	15	24.4%	-0.78 [-1.50, -0.06]
Jin 2013	18	4	20	15	4	20	25.0%	0.74 [0.09, 1.38]
Lim 2018	417	25	21	415	10	23	25.4%	0.10 [-0.49, 0.70]
Qiao 2015	49.1372	6.7044	30	38.3593	5.0919	30	25.3%	1.79 [1.18, 2.39]
**Total (95% CI)**			**88**			**88**	**100.0%**	**0.47 [-0.56, 1.51]**
Heterogeneity: Tau² = 1.01; Chi² = 31.55, df = 3 (P < 0.00001); I² = 90%								
Test for overall effect: Z = 0.89 (P = 0.37)								

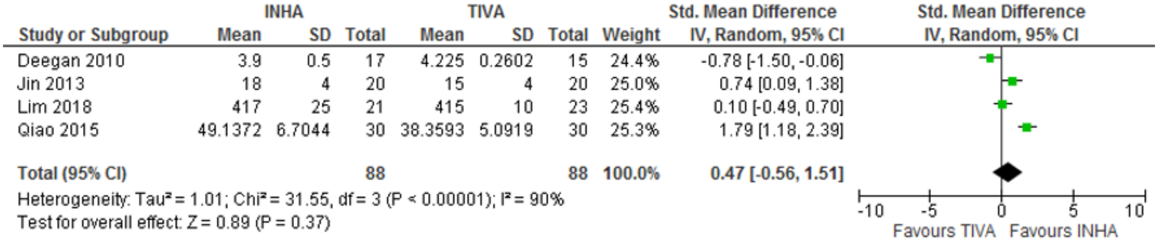

Supplementary Figure 9. Forest plot of TNF-α at 24 hours after surgery in TIVA and IHNA cohorts

Study or Subgroup	INHA Mean	INHA SD	INHA Total	TIVA Mean	TIVA SD	TIVA Total	Weight	Std. Mean Difference IV, Random, 95% CI
Deegan 2010	1,701.5	234.5	17	2,403.25	460.601	15	15.2%	-1.91 [-2.76, -1.05]
Jin 2013	20	7	20	22	7	20	16.5%	-0.28 [-0.90, 0.34]
Lim 2018	470	15	21	485	35	23	16.7%	-0.54 [-1.14, 0.06]
Margarit 2014	9.1	53.475	30	14	8.125	30	17.1%	-0.13 [-0.63, 0.38]
Oh 2018	610	23.3	102	610	38.3	99	18.0%	0.00 [-0.28, 0.28]
Tian 2017	187,116.6	18,404.9	31	153,374.2	11,119.7	31	16.5%	2.19 [1.55, 2.83]
**Total (95% CI)**			**221**			**218**	**100.0%**	**-0.09 [-0.89, 0.72]**
Heterogeneity: Tau² = 0.91; Chi² = 68.57, df = 5 (P < 0.00001); I² = 93%								
Test for overall effect: Z = 0.21 (P = 0.83)								

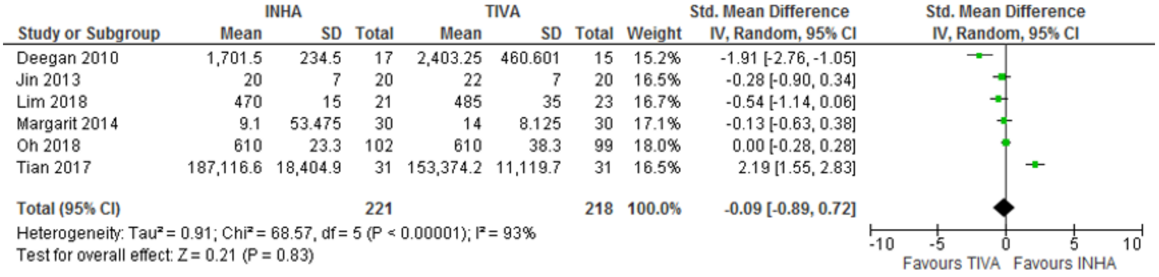

Supplementary Figure 10. Forest plot of IL-10 at 24 hours after surgery in TIVA and IHNA cohorts
